# Developing a novel dosiomics model to predict treatment failures following lung stereotactic body radiation therapy

**DOI:** 10.3389/fonc.2024.1438861

**Published:** 2024-12-12

**Authors:** Ashok Bhandari, Kurtis Johnson, Kyuhak Oh, Fang Yu, Linda M. Huynh, Yu Lei, Sarah Wisnoskie, Sumin Zhou, Michael James Baine, Chi Lin, Chi Zhang, Shuo Wang

**Affiliations:** ^1^ Department of Radiation Oncology, University of Nebraska Medical Center, Omaha, NE, United States; ^2^ Department of Biostatistics, University of Nebraska Medical Center, Omaha, NE, United States; ^3^ Department of Radiation Oncology, Novant Health Cancer Institute, Winston-Salem, NC, United States

**Keywords:** radiomics, dosiomics, lung SBRT, modeling, CT-dose interaction, treatment failure, Non-Small Cell Lung Cancer (NSCLC)

## Abstract

**Purpose:**

The purpose of this study was to investigate the dosiomics features of the interplay between CT density and dose distribution in lung SBRT plans, and to develop a model to predict treatment failure following lung SBRT treatment.

**Methods:**

A retrospective study was conducted involving 179 lung cancer patients treated with SBRT at the University of Nebraska Medical Center (UNMC) between October 2007 and June 2022. Features from the CT image, Biological Effective Dose (BED) and five interaction matrices between CT and BED were extracted using radiomics mathematics. Our in-house feature selection pipeline was utilized to evaluate and rank features based on their importance and redundancy, with only the selected non-redundant features being used for predictive modeling. We randomly selected 151 cases and 28 cases as training and test datasets. Four different models were trained utilizing the Balanced Random Forest framework on the same training dataset to differentiate between failure and non-failure cases. These four models utilized the same number of selected features extracted from CT-only, BED-only, a combination of CT and BED, and a composite of CT and BED including their interaction matrices, respectively.

**Results:**

The cohort included 125 non-failure cases and 54 failure cases, with a median follow-up time of 34.4 months. We selected the top 17 important and non-redundant features (with the Pearsons’s coefficient < 0.5) in each model. When evaluated on the same independent test set, the four models—CT features-only, BED features-only, a combination of CT and BED features, and a composite model including features from CT and BED that includes their interaction matrices—achieved AUC values of 0.56, 0.75, 0.73, and 0.82, respectively, with corresponding accuracies of 0.61, 0.79, 0.71, and 0.79. The composite model demonstrated the highest AUC and accuracy, indicating that incorporating interactions between CT and BED reveals more predictive capabilities in distinguishing between failure and non-failure cases.

**Conclusion:**

The dosiomics model integrating the interaction between CT and Dose can effectively predict treatment failure following lung SBRT treatment and may serve as a useful tool to proactively evaluate and select lung SBRT treatment plans to reduce treatment failure in the future.

## Introduction

Despite the decline in the rate of new cases and deaths in recent years (1), lung cancer remains the leading cause of cancer-related deaths in the US, accounting for over 20% of all cancer-related deaths (2). Non-small cell lung cancer (NSCLC) is the predominant type, accounting for more than 80% of all lung cancers (3). With the rapid advances in lung cancer screening (4), NSCLC is increasingly diagnosed at earlier stages (3). Although surgery remains the standard of care for early-stage lung cancer (5), stereotactic body radiation therapy (SBRT) has rapidly been accepted as a standard treatment for patients with early-stage medically inoperable NSCLC (6–8). SBRT allows delivering ablative radiation dose to the target while maintaining tolerable toxicities (9–12) by utilizing modern techniques in motion management (13–16) and imaging guidance technologies (17, 18). Despite achieving an excellent local control rate, lung SBRT still exhibits a relatively high total failure rate, combining both regional and distant failures, at approximately 30% (6, 7). Novel treatment assessment technologies are still warranted to predict potential treatment failures, thereby improving the survival benefits.

Radiomics is a machine learning-based quantitative image analysis technology. Owing to its distinct advantages in providing a comprehensive and quantitative representation of the radiographic phenotype of a 3D target volume (19–25), it has become an active area of research for risk assessment and treatment response prediction in cancer management (26–30). However, radiomics is still limited in comprehensive treatment response assessment, especially in radiation oncology, because it only extracts and analyzes features from the medical images without considering radiation dose. Dose-volume histograms (DVH) are the standard metric in radiation oncology to assess dose coverage to the target and dose exposure to the organs at risk (OARs), assisting in the prediction of treatment outcomes and potential toxicities. However, DVHs do not possess spatial information concerning the dose distribution in a 3D organ or lesion. Limited studies have examined how the subtle difference in the 3D spatial distribution of the radiation dose impacts the treatment response for similar treatments; these types of studies are categorized as dosiomics. First cited in 2018, the term dosiomics refers to studies that attempt to combine radiomics predictive mathematics with 3D spatial dose information (31). These existing dosiomics studies (32), however, only investigated the dose distribution without considering the interplay between the dose and the specific tissue irradiated. Theoretically, the interaction between radiation and the irradiated tissue, as represented by medical images, essentially influences the treatment outcome.

In this study, we designed a novel dosiomics model incorporating the interplay between tissue density and the biologically effective dose to predict potential treatment failure.

## Materials and methods

### Ethical approval and patient selection

**Figure 1:**
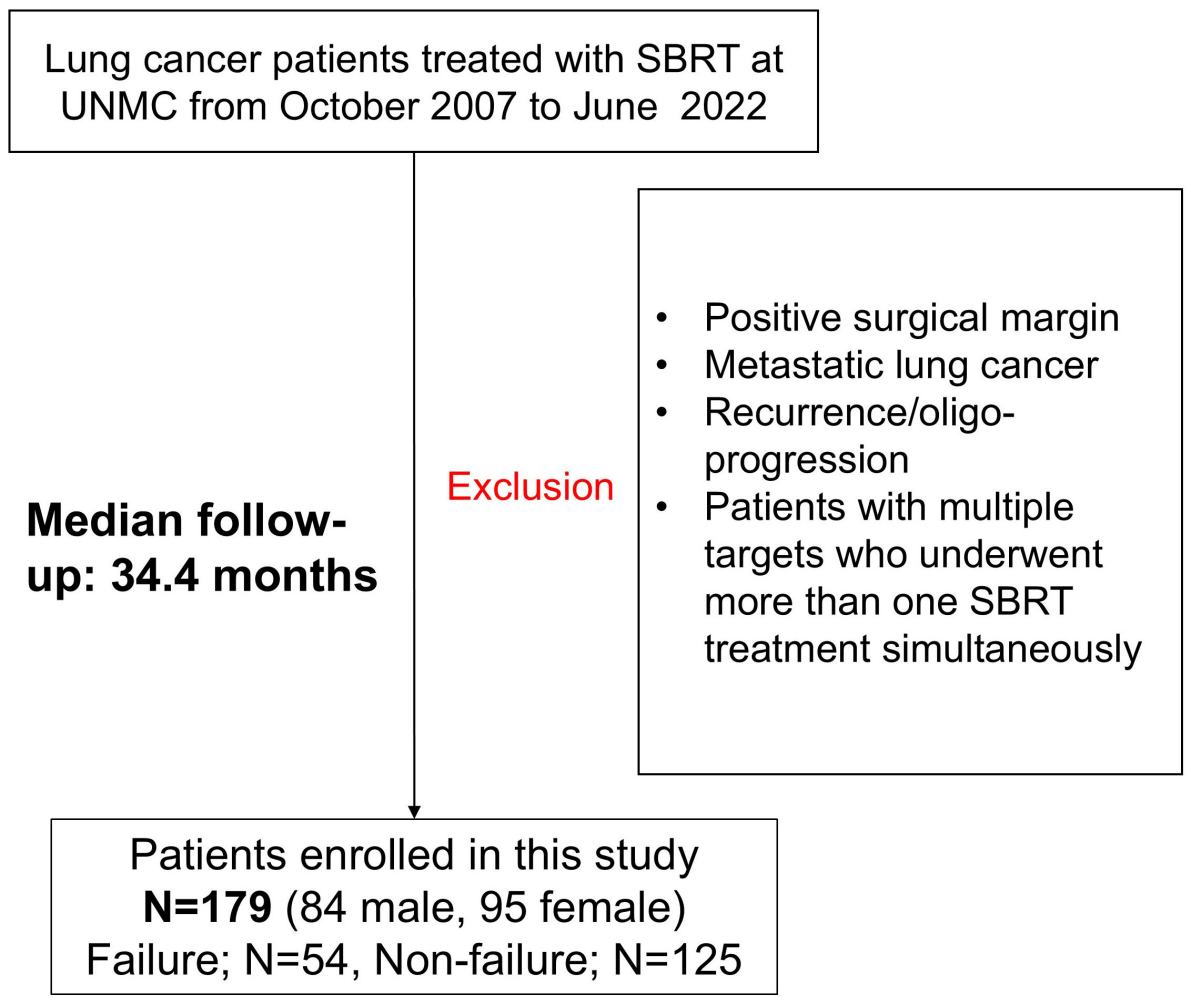
Flowchart of patient selection process for lung dosiomics study.

The Institutional Review Board at our institution approved this retrospective study (IRB 722-19-EP). We retrospectively enrolled consecutive NSCLC lung cancer patients treated with stereotactic body radiation therapy (SBRT) at the University of Nebraska Medical Center (UNMC) between October 2007 to June 2022. We exclusively selected cases determined to be the primary NSCLC based on multidisciplinary tumor board discussion about patient clinical history, imaging, and available pathology before radiation treatment. We excluded patients with the following conditions: 1) adjuvant treatment for positive surgical margins, 2) SBRT for metastatic disease, 3) recurrence/oligo-progression of previously treated primary lung tumor, and 4) patients with multiple targets who underwent more than one SBRT treatment simultaneously. In addition, we excluded patients with a follow-up time of less than a year, except those patients who presented with treatment failures between 6 months to a year after the SBRT treatment. **Figure 1** summarizes the patient selection process of our study. As summarized in **Table 1**, the patient cohort included 84 male and 95 female patients. The median age is 73 in the range from 52 to 91. The median follow-up time is 34.4 months, ranging from 7.3 to 178.5 months, with a 10-90 percentile range of 73.5 months. 81 cases are adenocarcinoma, 60 cases are squamous cell carcinoma, 9 cases are NSCLC (not otherwise specified - NOS), and 2 cases are large cell carcinoma. The cohort also included 27 presumed NSCLC cases without tissue confirmation. After the medical record review, 125 cases are considered as non-failure cases, and 54 cases are determined to have treatment failure.

**Table 1:** Patient characteristics.

Patient Characteristics	
Gender	
Male	84
Female	95
Follow-Up (months)	
Median	34.4
Range	7.3 -178.5
PTV (cc)	
Mean ± STD	33.1 ± 27.4
Histology	
Adenocarcinoma	81
Squamous Cell Carcinoma	60
NSCLC NOS	9
Large Cell	2
Presumed NSCLC	27
Tumor location	
RUL	45
RML	7
RLL	46
LUL	58
LLL	23
Fractionation	
1000x5	126
1200x4	25
1250x4	14
1800x3	7
1200x5	4
750x8	2
700x10	1

### Clinical endpoints

Treatment failure in this study includes local, regional, and distant failures. Local failure refers to the recurrence of cancer within the same lobe as the primary site of disease. Regional failures pertain to regional nodal recurrence, while distant failure involves recurrence in a different lobe or in extrathoracic regions. These definitions of treatment failures are consistent with RTOG0236. Local failures were assessed using PET imaging and biopsy, when deemed safe. If a biopsy was not feasible, we relied on multidisciplinary discussions based on at least two consecutive scans to determine if a case represented a true local failure. Regional failures were similarly confirmed through PET and involved endobronchial ultrasound with biopsy confirmation. Distant failures were validated using PET scans and biopsies, except in cases of brain metastases, which were not always biopsied. We also have closely examined the medical record for the non-failure cases, with standard care being chest CT every 3-4 months during the follow-up time within two years after SBRT. All available CTs right before the death were reviewed in the process.

### CT simulation

All the patients underwent a free-breathing computed tomography (CT) scan (FB-CT) and a 4D-CT scan using one of the three Siemens scanners: Sensation Open, Definition, or Confidence (Siemens Medical Solutions USA, Inc., PA, USA). All the scans were acquired using 120kVp with a slice thickness of 2 or 3 mm. The tube current used for the scans were ranging from 30 mA to 381 mA. Additionally, all images were acquired using the smooth convolution kernel equipped on the Siemens CT scanners (B31s or B31f).

### Target segmentation and volumes of interest

**Figure 2:**
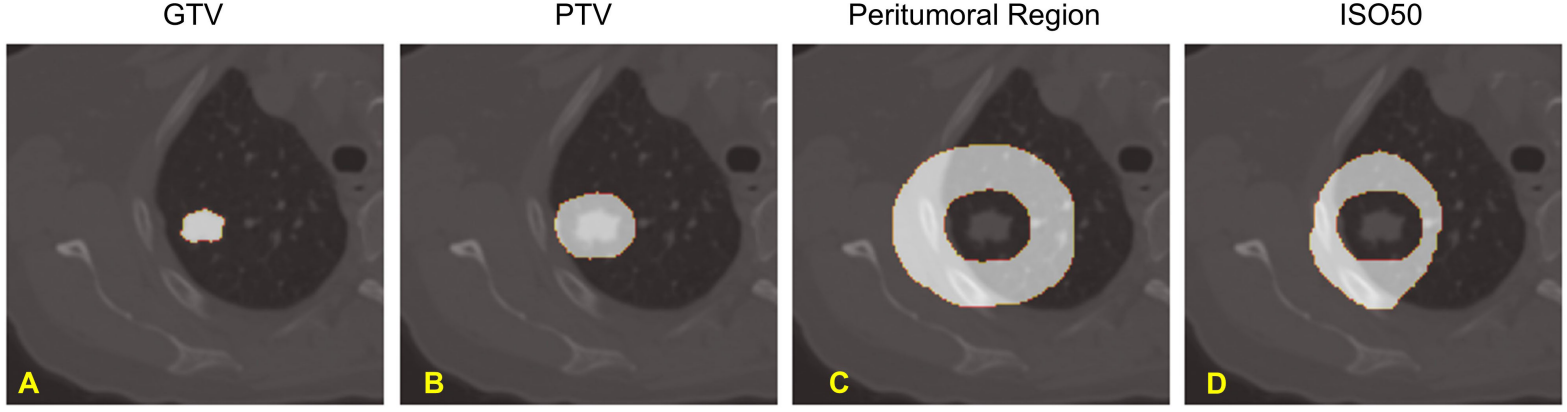
Volumes of Interest of our study: **(A)**. Gross Tumor Volume (GTV), **(B)**. Planning Target Volume (PTV), **(C)**. Peritumoral Region, and **(D)**. Normal Tissue receiving greater than 50% of Prescription Dose - ISO50.

The Gross Tumor Volume (GTV) and the Planning Target Volume (PTV) were delineated by the attending radiation oncologist based on the maximum intensity projection from the 4D CT scan during the treatment planning. To reduce the inter-observer variability, we created a semi-automatic GTV that encompasses the voxels with the HU value between (-550 to 2000) within the clinical GTV volume. We also defined a peritumoral region that included an outer 2 cm ring outside the PTV and a dose structure (ISO50) for the tissues that received equal to or more than 50% of the prescription dose outside the PTV. Our rationale to create these volumes of interest outside PTV is that peritumoral radiomics signatures have been demonstrated to be valuable in cancer prognosis assessment (27). In addition, the peritumoral region outside the PTV typically experiences a rapid dose fall-off in Lung SBRT, resulting in a notably nonuniform dose distribution across this area. The volumes of interest of an example patient were shown in **Figure 2**.

### Dose calculation

The treatment plans of 133 patients were created on the free breathing (FB) CT whereas the remaining 46 patients were planned on the average intensity projection (average CT) from their respective 4DCT scans. To ensure consistency among the plans in this study, all the plans with the radiotherapy structures that were created on the average CT images were copied to their respective FBCT images, and the radiotherapy plan was recalculated with the same monitor units (MU) without re-optimizing. Among the 179 patients, 3 patients were planned using the collapsed-cone-convolution superposition (CCC) algorithm in the Pinnacle treatment system (TPS) (Philips Medical Systems, Fitchburg,WI). 23 patients were planned in the iPlan treatment planning system (Brainlab AG, Feldkirchen, Germany). The remaining patients were planned the Analytical Anisotropic Algorithm (AAA) in Eclipse treatment planning system (Varian Medical Systems, Palo Alto, CA). For the 23 patients planned in iPlan, the machine was recommissioned in the Eclipse TPS, and the plans were recalculated by the AAA algorithm for improved heterogeneity correction.

### Fractionation

The fractionation schemes are summarized in **Table 1**. 126 patients received 1000cGy x 5 fractions, 25 patients received 1200cGy x 4 fractions, 14 patients received 1250cGy x 4 fractions, 7 patients received 1800cGy x 3 fractions, 4 patients received 1200cGy x 5 fractions, 2 patients received 750cGy x 8 fractions and 1 patient received 700cGy x 10 fractions.

### Data preprocessing

The CT volume images, target segmentation, and 3D dose distribution matrix of each patient were exported in DICOM format in Eclipse TPS. We converted all the DICOM files to NRRD format using a batch process in 3D slicer software (33) https://www.slicer.org/), as previously described (34, 35). We then resampled volume CT images, 3D dose distribution matrices, and the target segmentations with a 1 x 1 x 2mm^2^. Additionally, we performed cropping on all matrices using the segmentation mask encompassing the peritumoral region, along with a margin in all three directions. This process ensured that all matrices of a patient possess identical dimensions and resolution.

### Voxelated biological effective dose

We used a linear quadratic (LQ) radiobiological model to generate a 3-D distribution of Biologically Effective Dose (BED) from the 3D physical dose distribution. The voxels within the PTV are considered cancerous and are assigned with an α/β ratio of 10Gy. The voxels outside the PTV are considered healthy tissue, and hence were assigned with an α/β ratio of 3Gy. Depending on the α/β ratio for each voxel, the corresponding BED is calculated using Equation 1, as shown below:


(1) $$BED_{i,j,k}\;=\;n\;\times \;d_{i,j,k}\;\left(1\;+\;\frac{d_{i,j,k}}{{\left(\frac{\alpha }{\beta }\right)}_{i,j,k}}\right)\;$$

where BED_i,j,k_ is the BED for the voxel at position (i,j,k), n is the number of fractions, *d_i,j,k_* is the dose per fraction at voxel (i,j,k), and ${\frac{\alpha }{\beta }}_{i,j,k}$ is the alpha-beta ratio (10 for cancerous tissue or 3 for normal tissue) for the voxel at position (i,j,k) depending on if the voxel is inside or outside the PTV.

### Calculation of the voxelated interaction matrices

**Figure 3:**
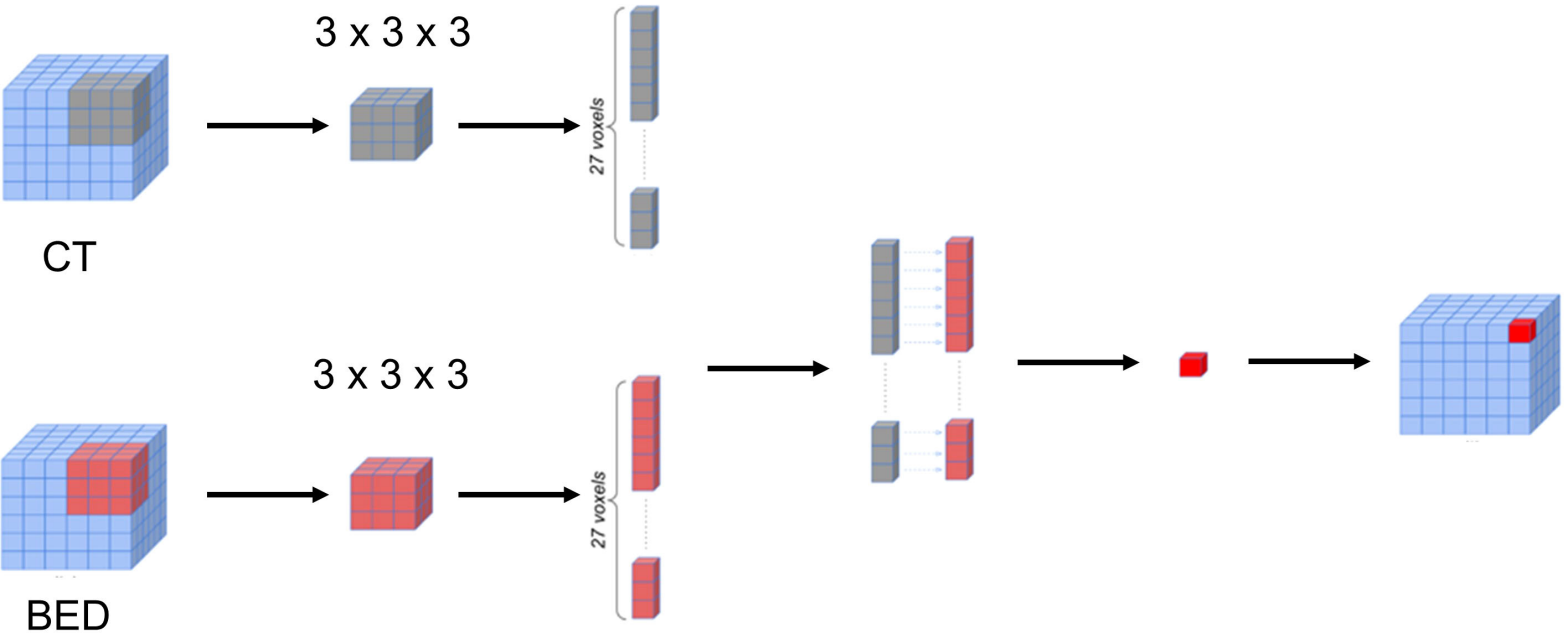
Schematic representation of the calculation of the voxelated interaction matrices.

The BED matrix and the planning CT image matrix exhibit a point-to-point correspondence. Specifically, each voxel within the BED matrix corresponds to a voxel within the CT image matrix with the same coordinates, representing the dose received by the tissue in the corresponding voxel of the CT image matrix. To delve deeper into the potential features concealed within the relationship between the CT image matrix and the BED matrix, we generated a series of interaction matrices to depict the interrelationship between the CT image and the Biological Effective Dose (BED). Specifically, we extracted a pair of three-dimensional patches (3 x 3 x 3) surrounding each voxel in both the CT image matrix and BED matrix. Subsequently, we computed various metrics, including Entropy, Spearman Rank Coefficient, Jensen-Shannon Divergence (JSD), and Wasserstein distance between these two patches (CT and BED). Each of these metric values was then recorded within an empty matrix, preserving the spatial alignment with the original voxel location within the CT image matrix. **Figure 3** illustrated the process of calculating the 3-D interaction matrices between CT image and BED. In addition, we also calculated a pair-wise multiplication matrix between the CT image and the BED dose matrix.

### Feature extraction

PyRadiomics (24), an open-source package, was used to extract radiomics features. Briefly, we converted the DICOM images and target delineation to NRRD format using a batch process in 3D slicer software (33) https://www.slicer.org/), as previously described (34, 35).

### Data split for training and testing

We randomly split the dataset into training and test subsets, comprising 84% (151 cases) and 16% (28 cases) of the total data, respectively. Both datasets were balanced to maintain similar ratios of failure and non-failure cases.

### Feature selection

Following the feature extraction, we selected the important and non-redundant features from the features extracted from the CT image, BED and the calculated interaction matrices using our in-house feature selection pipeline that has been previously reported (34, 35). The feature selection pipeline consists of two main steps: quantifying feature importance and identifying non-redundant features. Feature importance is determined through repeated 5-fold cross-validation using various classification algorithms from the training dataset (151 cases), including Random Forest, Extra Trees, AdaBoost, and XGBoost. The final importance score for each feature is the average of the scores from all algorithms. Pearson’s correlation coefficient was then calculated among these features in descending order to identify non-redundant features, with the top-ranked feature automatically considered non-redundant. For each subsequent feature, if the Pearson coefficient with any previously identified non-redundant feature exceeds 0.5, it is deemed redundant and discarded.

### Classification predictive modeling

Four different models were trained utilizing the Balanced Random Forest (BRF) framework on the same training dataset (151 cases) to differentiate between failure and non-failure cases. These models utilized various combinations of features: CT-only, BED-only, a combination of CT and BED, and a composite of CT and BED including their interaction matrices, respectively. We utilized a grid-search approach to optimize the hyperparameters of the BRF classifier. We explored a parameter grid consisting of ‘‘max_depth’’, ‘‘n_estimator’’, ‘min_samples_split’, ‘‘min_samples_leaf’’, ‘‘max_feature’’, ‘‘bootstrap’’ and ‘‘criterion’’ with specific value ranges. The parameters are summarized in **Table 2**. We created a BRF classifier with the defined hyperparameters and utilized the GridSearchCV to perform the grid search by applying the 5-fold cross-validation on the training set. We constructed the BRF classifier model with the best hyperparameters identified in the grid search and tested the model performance on the test dataset.

**Table 2:** Summary of the search ranges for each parameter during hyperparameter tuning of the Balanced Random Forest (BRF) classification model.

Model hyperparameter name	Search space for optimal hyperparameter
n_estimators	50, 100, 200, 300
max_depth	2, 3, 5, 7
min_samples_split	2, 4, 6, 8, 10
min_samples_leaf	1, 2, 3
max_features	auto, sqrt, log2
bootstrap	True, False
criterion	gini, entropy

### Model performance evaluation

The performance of the models was quantified by the area under the receiver operating characteristic curve (AUC), accuracy, sensitivity, and specificity. Besides these metrics, we implemented Delong test (36) to compare the AUCs from different models. We also conducted Decision Curve Analysis (DCA) to evaluate the clinical utility of our predictive models. DCA assesses the net benefit of the model by considering true positives and false positives across a range of threshold probabilities, reflecting the trade-off between interventions and their potential outcomes. We calculated net benefit using standardized formulas, comparing the model’s performance to both default strategies—assuming all patients would receive intervention and assuming none would in Python (37). DCA plots were generated to visually represent the model’s clinical impact, enabling an assessment of its value in guiding patient decision-making. Additionally, we plotted the calibration curve and calculated the Brie score for each model. Briefly, calibration curves were computed using scikit-learn package in Python, providing mean predicted probabilities and corresponding fractions of positive outcomes. Brier score losses, from scikit-learn, were also calculated to quantify prediction accuracy. We visualized the calibration curves for all models on a single plot.

### Competing risk time-to-event analysis

The Fine-Gray model (38) was used to predict the cumulative incidence of having treatment failure using the features identified by the four considered models, including CT-only, BED-only, a combination of CT and BED, and a composite of CT and BED including their interaction matrices, separately. The concordance index (c-index) was estimated to assess the models’ ability to separate individuals with treatment failure earlier than others. The c-index value ranges between 0 and 1, and a value of 0.5 suggests no discriminating ability, and a value of 1 suggests perfect discriminating ability. The Brier score was evaluated to assess both calibration (i.e. how close the prediction is to the true underlying risk of event) and discrimination. A smaller Brier score indicates a better model. The integrated Brier score (IBS) was calculated to integrate the Brier score values obtained at all follow up times to assess the overall performance of the considered models. The competing risk analyses using the prodlim, riskRegression, and pec packages of R statistical software (R version 4.4.1, http://www.r-project.org).

## Results

### BED and interaction matrices

**Figure 4:**
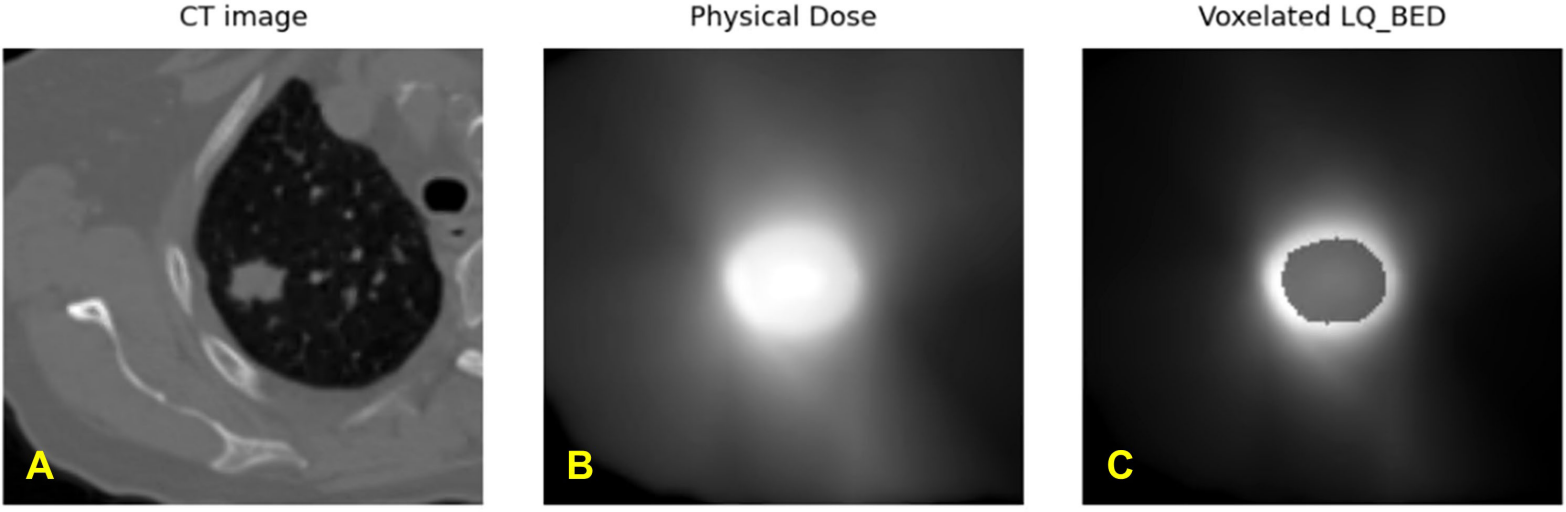
Exemplary representation of **(A)** planning CT image, **(B)** physical dose distribution extracted directly from the treatment plan, and **(C)** calculated biologically effective dose (BED) distribution from one patient.

**Figure 5:**
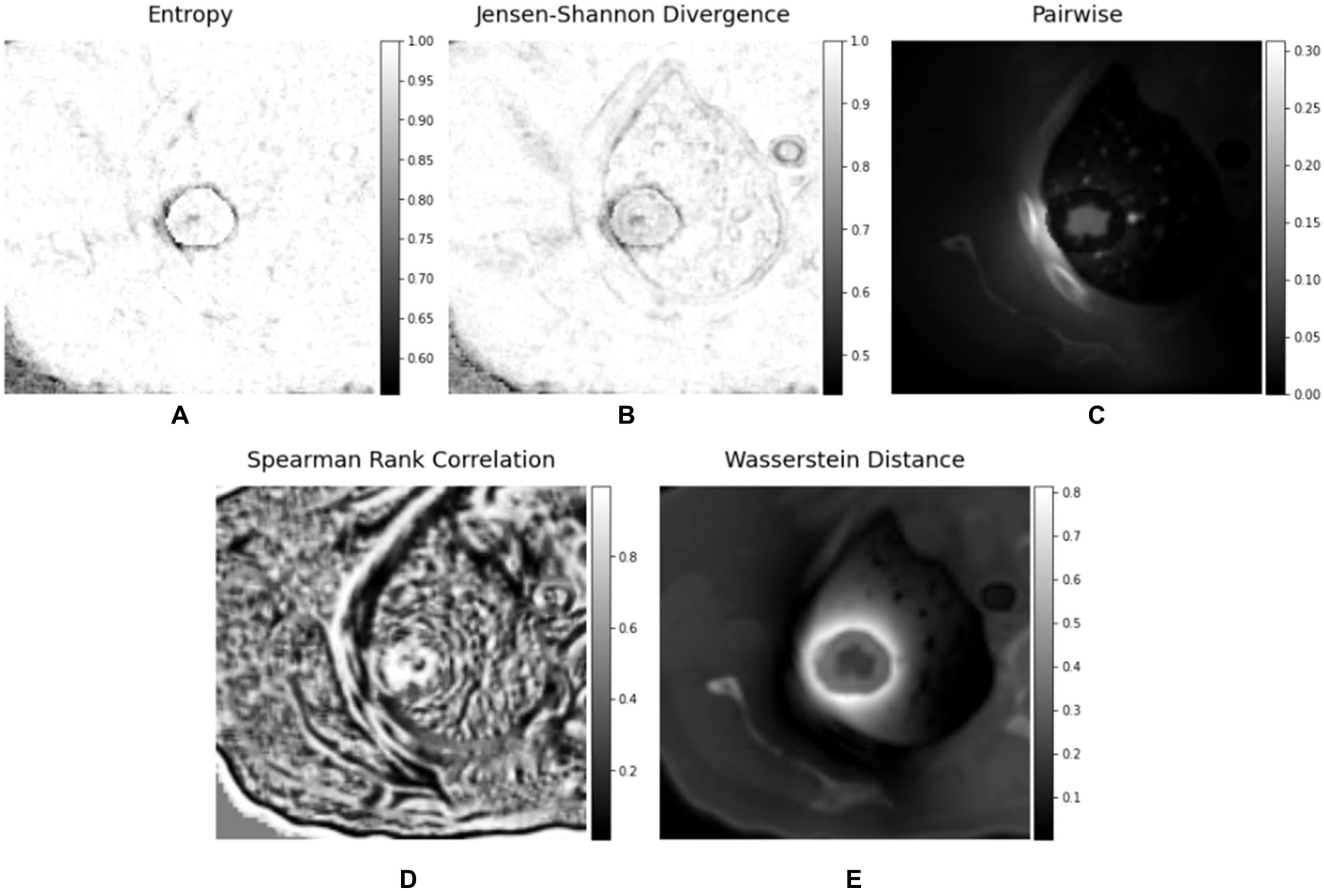
Interaction matrices calculated using planning CT and biologically effective dose (BED) - **(A)** Entropy matrix, **(B)** Jensen-Shannon Divergence matrix **(C)** pairwise multiplication matrix, **(D)** Spearman rank correlation matrix, and **(E)** Wasserstein distance matrix.

We have calculated the biologically effective dose (BED) distribution from the physical dose distribution using a linear quadratic (LQ) radiobiological model as described above. **Figure 4** showed an example of a planning CT, a physical dose distribution extracted directly from the treatment plan, and the corresponding biologically effective dose (BED) distribution. **Figure 5** showed interaction matrices calculated using planning CT and biologically effective dose (BED) distribution. (A) the Entropy matrix, (B) the Jensen-Shannon Divergence matrix (C) the pairwise multiplication matrix, (D) the Spearman rank correlation matrix, and E) the Wasserstein distance matrix.

### Feature extraction

We extracted features from the four volumes of interest (GTV, PTV, peritumor region, and ISO50) on each set of the seven matrices of interest – the CT image matrix, LQ_BED matrix, and five interaction matrices. From each ROI in a single matrix, 105 features were extracted, including 14 shape-based features and 91 first-order and textual features. Using radiomics mathematics, features were extracted from the volumes of interest (GTV, PTV, Peritumoral Region, and ISO50) across various matrices (CT, BED, Entropy, Jensen-Shannon Divergence, pairwise multiplication, Spearman rank correlation, and Wasserstein distance matrices). This process resulted in 420, 420, 840, and 2940 features, respectively, which were then processed in our feature selection pipeline, and the selected features were then utilized to develop four predictive models: CT-only, BED-only, a combination of CT and BED, and a composite model of CT and BED that integrates their interaction matrices.

### Feature selection

**Figure 6:**
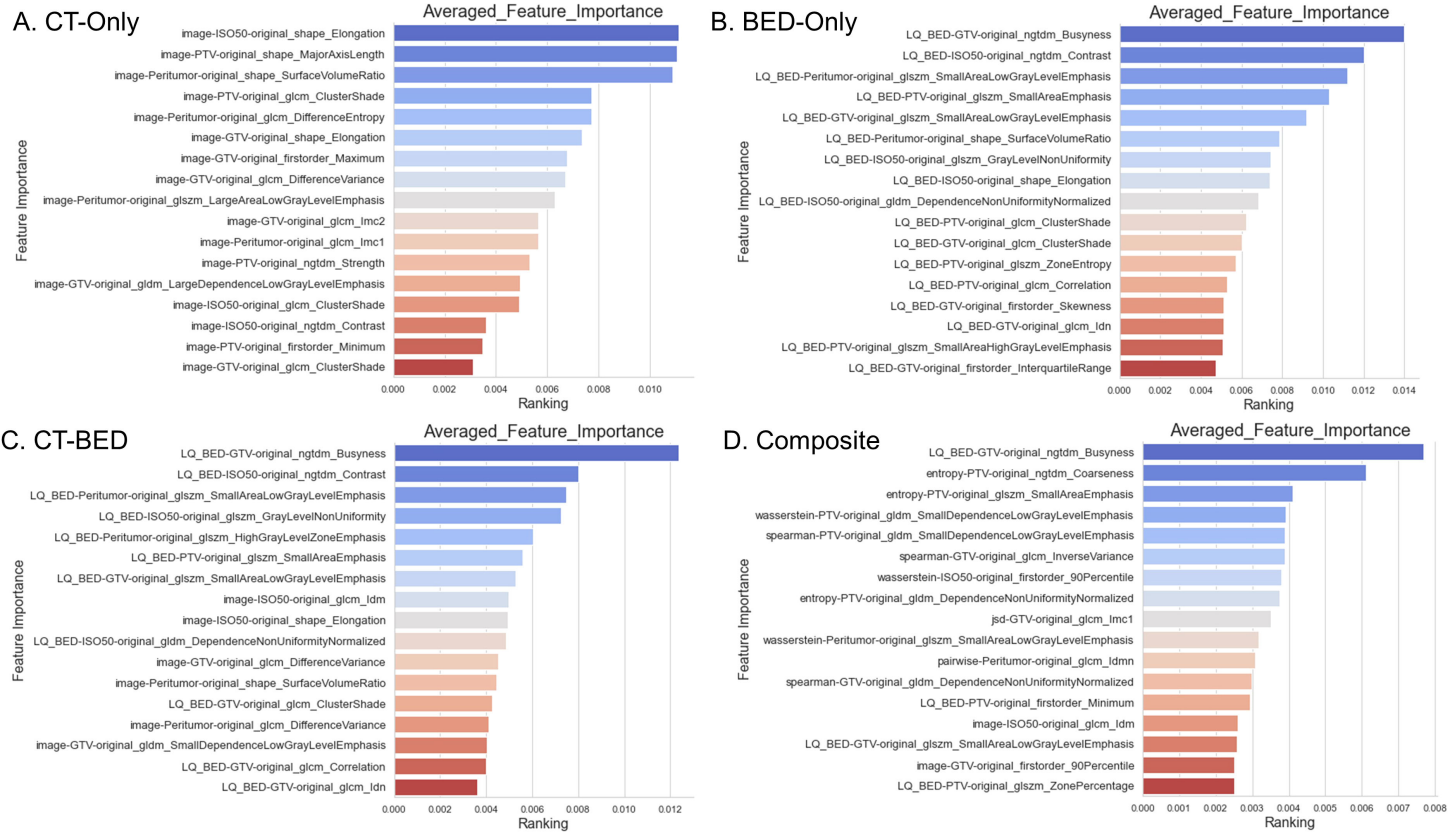
Feature importance of the four models: **(A)** CT-Only model; **(B)** BED-Only model; **(C)** CT-BED model; **(D)** Composite model.

**Figure 7:**
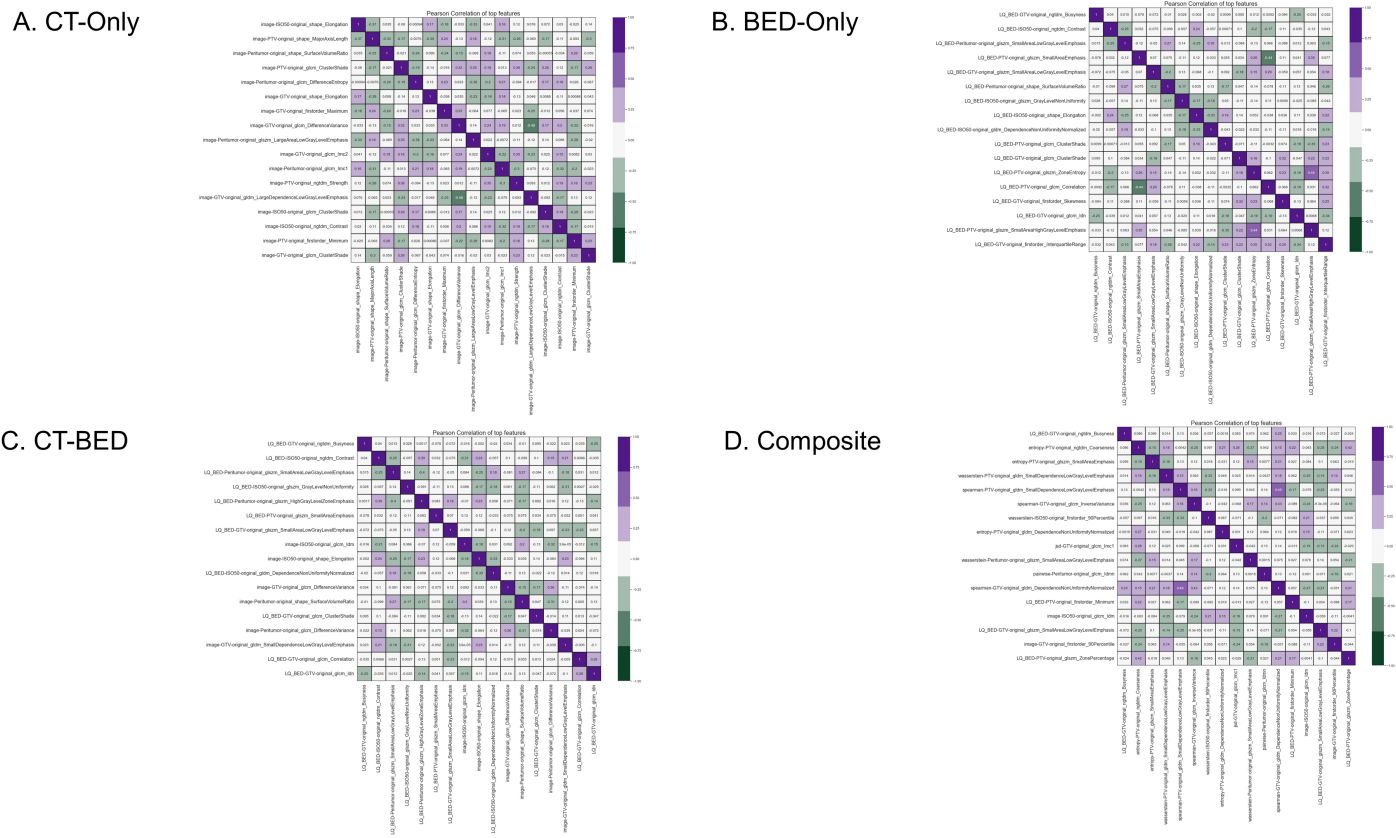
Pearson coefficient of features for **(A)** CT-Only, **(B)** BED-Only, **(C)** CT-BED, and **(D)** composite model.

From the same training set (151 patients), our in-house feature selection pipeline resulted in 17 important and non-redundant features for each model. The importance ranking is shown in **Figure 6**, and non-redundancy, assessed by the Pearson Coefficient being less than 0.5, are shown in **Figures 7A–D**, respectively.

### Model performance

**Figure 8:**
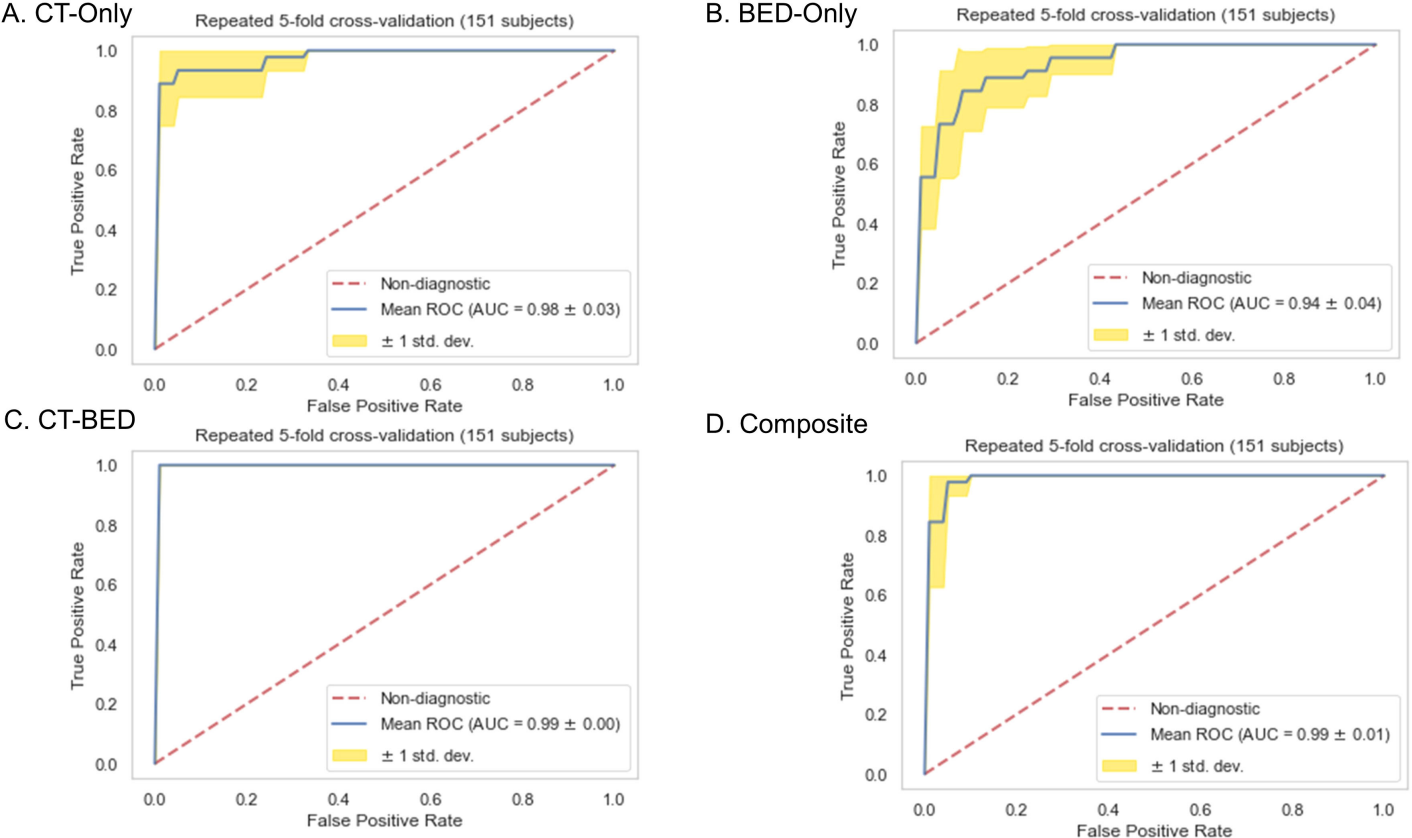
Receiver-operating characteristic (ROC) curves of **(A)** CT-Only, **(B)** BED-Only, **(C)** CT-BED, and **(D)** composite model on the same training set.

**Figure 9:**
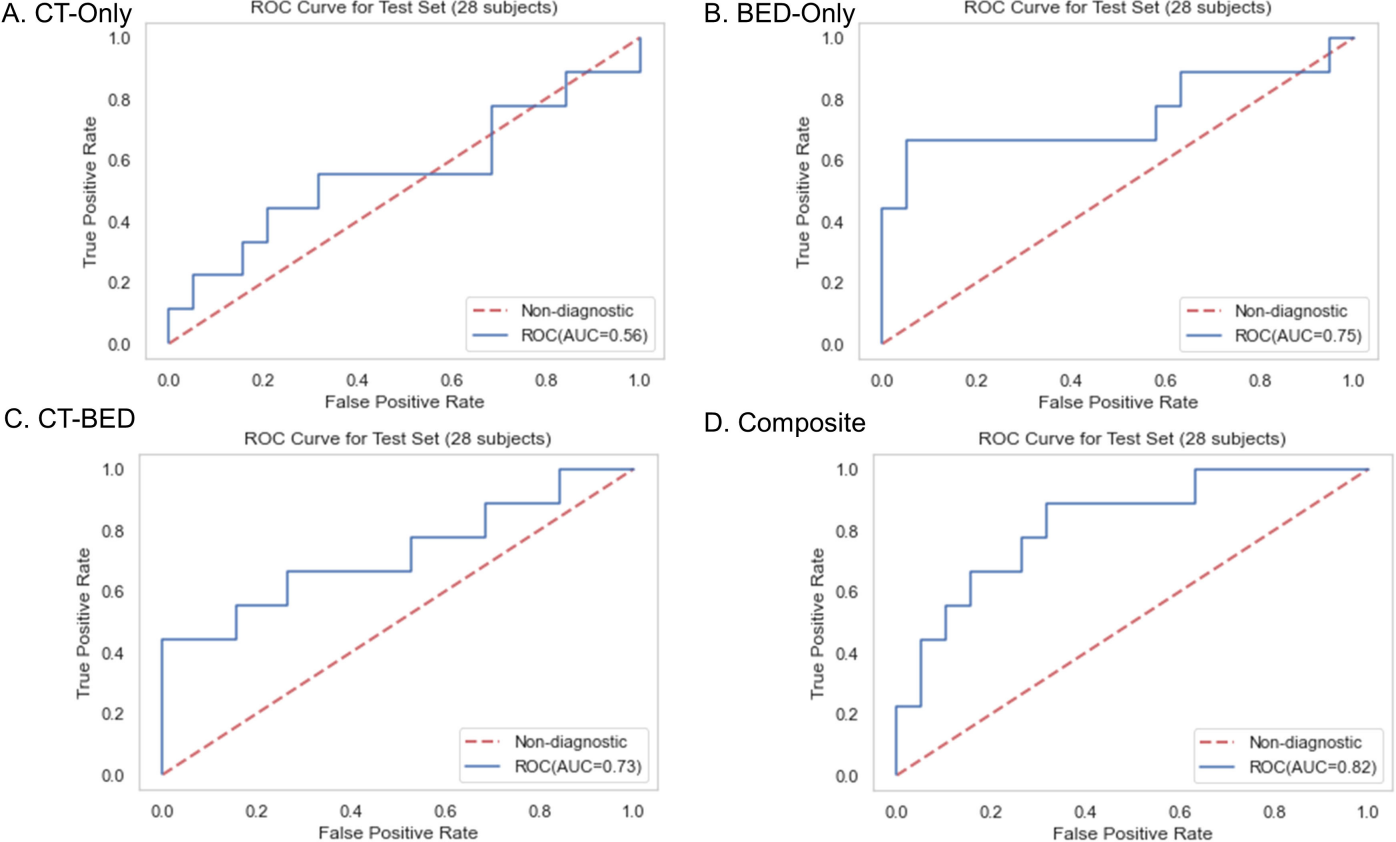
Receiver-operating characteristic (ROC) curves of **(A)** CT-Only, **(B)** BED-Only, **(C)** CT-BED, and **(D)** composite model on the same independent test set.

**Figure 10:**
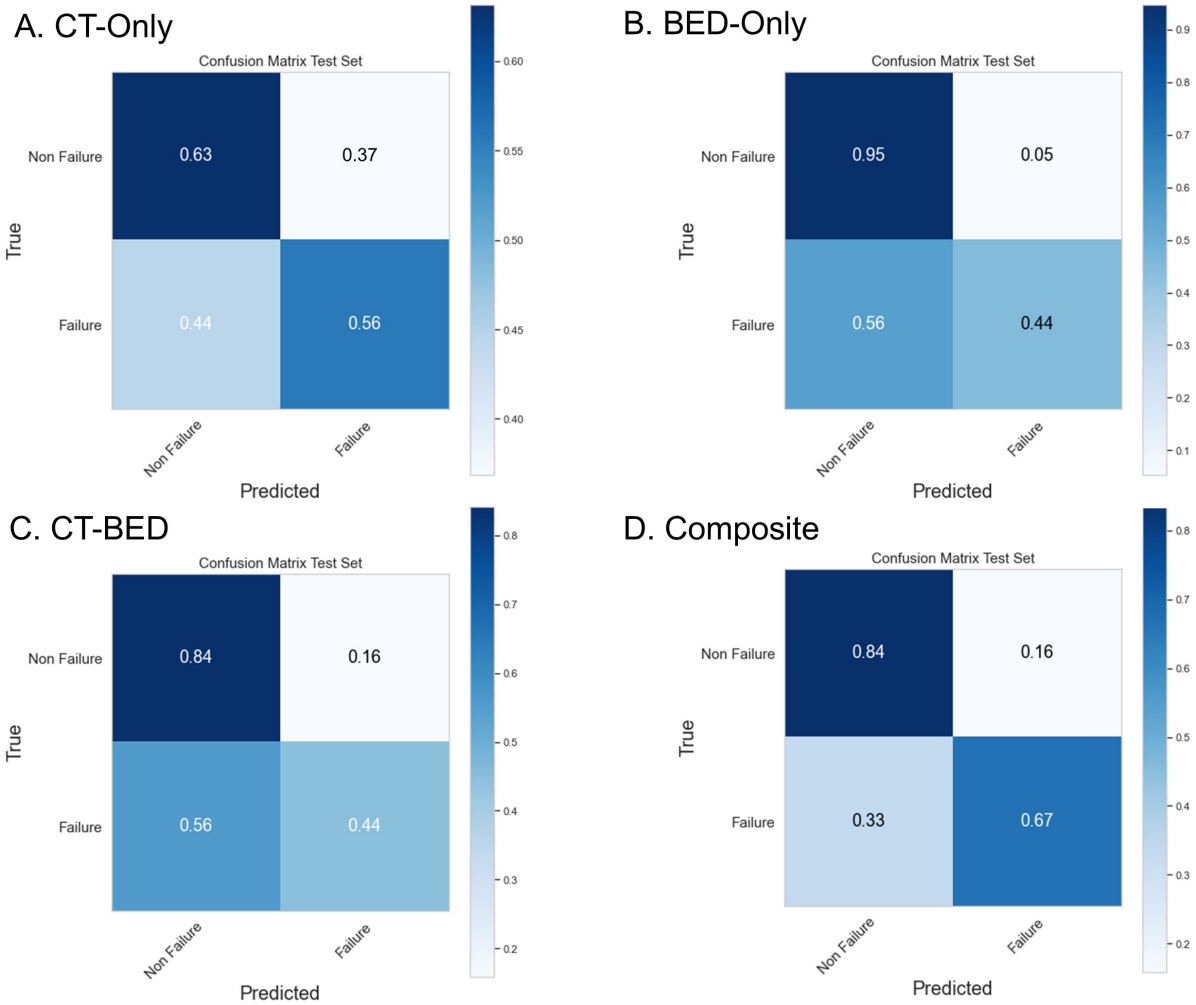
Confusion Matrix of **(A)** CT-Only, **(B)** BED-Only, **(C)** CT-BED, and **(D)** composite model on the same independent test set.

Repeated 5-fold cross-validation on the training dataset comprising 151 subjects demonstrated that the Balanced Random Forest model attained an average area under the receiver operating characteristic curve (AUC) of 0.98, 0.94, 0.99, and 0.99, respectively (**Figure 8**). Upon evaluation with the same independent testing set of 28 subjects, each model achieved AUC values of 0.56, 0.75, 0.73, and 0.82, respectively, with corresponding accuracies of 0.61, 0.79, 0.71, and 0.79, as shown in **Figures 9A–D**). Sensitivity and specificity of each model were displayed in **Figures 10A–D** via confusion matrices. The p-values for each pairwise comparison between models, calculated using the Delong test, are listed in **Table 3**.

**Table 3:** Comparison of AUC values between different models using a Delong test.

	AUC	p-value
**CT vs BED**	0.56 vs 0.75	0.24
**CT vs CT-BED**	0.56 vs 0.73	0.30
**CT vs Composite**	0.56 vs 0.82	0.07
**BED vs CT-BED**	0.75 vs 0.73	0.78
**BED vs Composite**	0.75 vs 0.82	0.39
**CT-BED vs Composite**	0.73 vs 0.82	0.34

P-value >0.05 suggests that the difference between the area under curve (AUC) are not significantly different.

**Figure 11:**
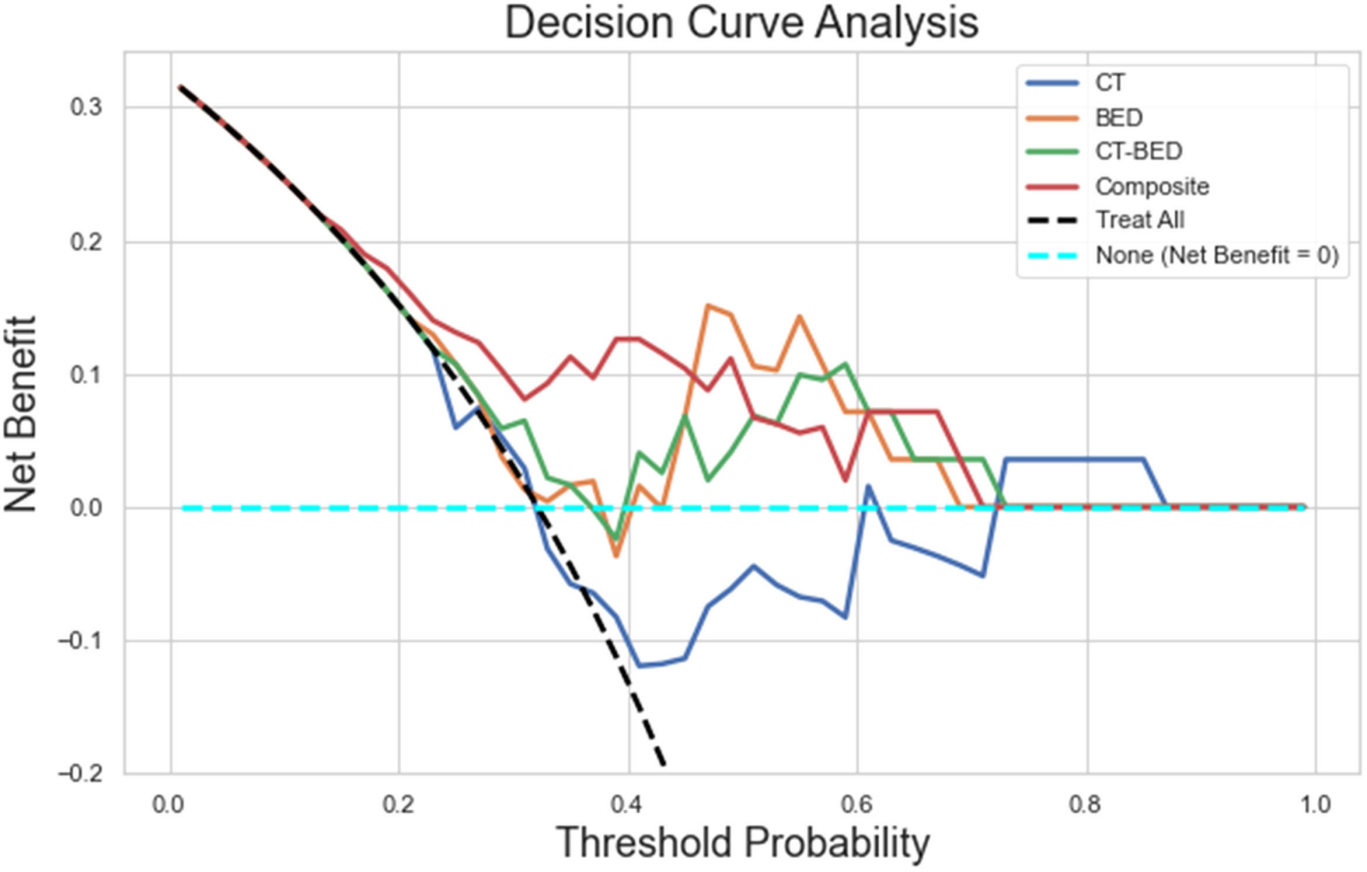
The decision curve analysis plot illustrating the net benefit of four models (CT, BED, CT-BED, and Composite) compared to the "Treat All" and "Treat None" scenarios across various threshold probabilities.

**Figure 12:**
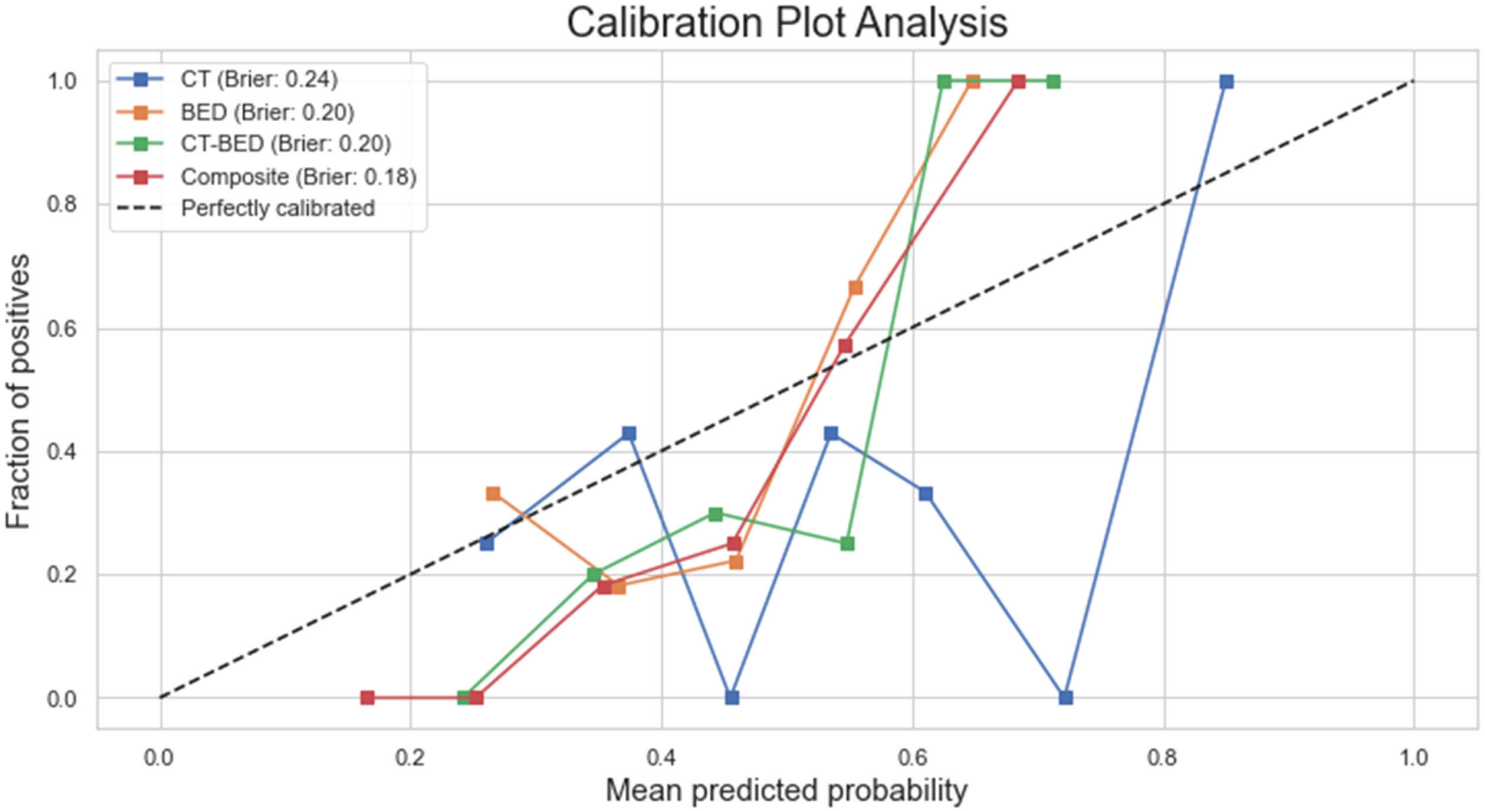
Calibration plot for each model with their brier scores.

As shown in **Figure 11**, the Decision Curve Analysis plot illustrated the net benefit of four models (CT, BED, CT-BED, and Composite) compared to the “Treat All” and “Treat None” scenarios across various threshold probabilities. The Composite model (red line) showed the highest net benefit over a wide range of threshold probabilities, suggesting it provides the most clinical utility. The other models, on the other hand, demonstrated fluctuating net benefits, with the CT-BED (green line) also performing well at certain thresholds. Our results indicated that the Composite model may be the most effective for guiding clinical decisions within the threshold probability range shown. As shown in **Figure 12**, the composite model achieved a Brie score of 0.18 indicating its superiority compared to other models in reflecting the observed outcomes by its predicted probabilities. The Brie score for both BED and CT-BED models are 0.20 and 0.20, respectively, showing that they had more deviations from the perfect predictions compared to the composite model. The CT model, on the other hand, exhibited poor calibration, diverging from the diagonal line, suggesting that its probability estimates were less reliable.

### Competing risk time-to-event analysis

**Figure 13:**
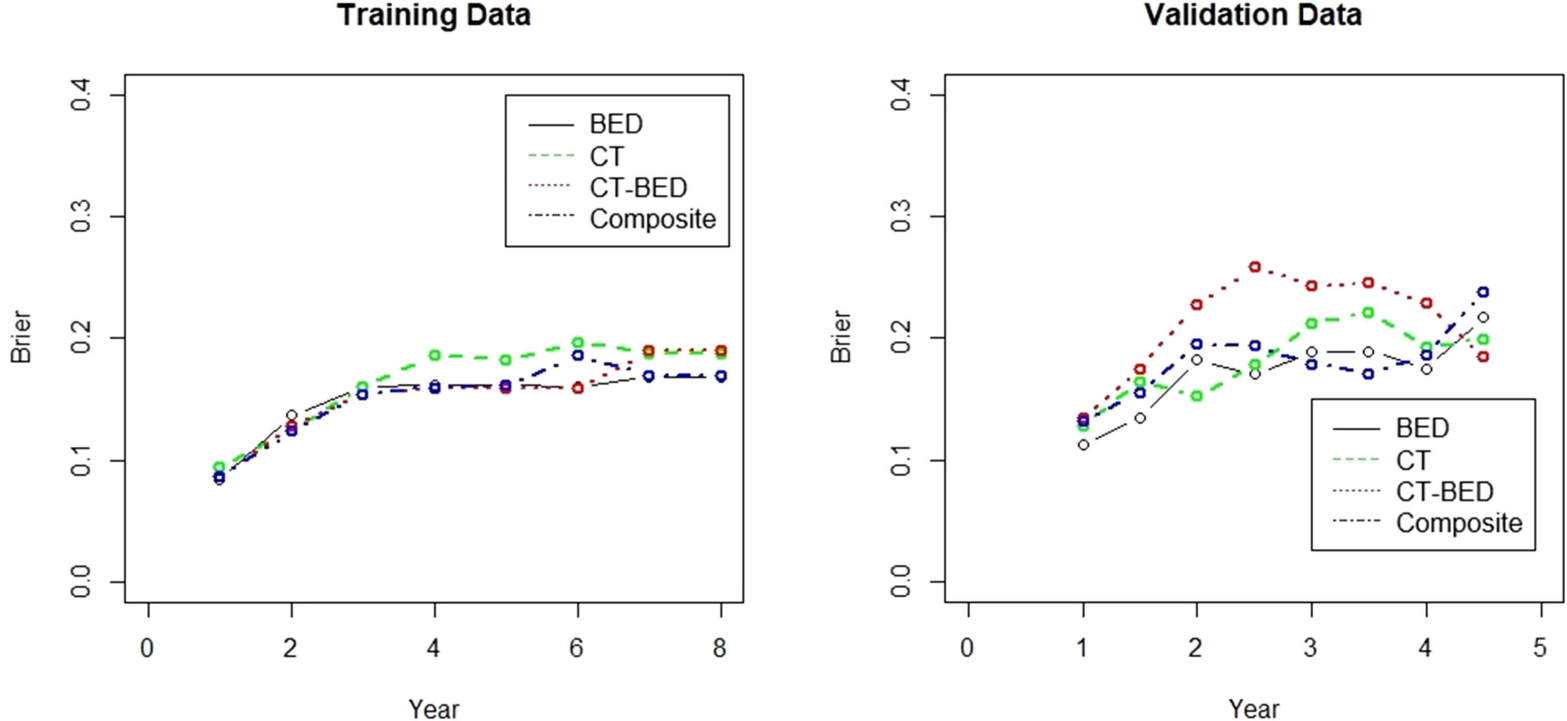
Brier score for the training and validation data for the competing risk analysis.

**Figure 13** showed the Brier score for the training and validation data, respectively. There is no treatment failure or competing death event after 8 years for the training data and 4.5 years for the test data following the treatment. Therefore, a time range of 1 year to 8 years after treatment was used for the plot on training data and 1 year to 4.5 years for the plot of the validation data. For the training data, the Brier scores are similar and less than 0.25 for all considered models at year 1 to year 8, indicating all these considered models provides good performance. For the validation data, all models have Brier scores less than 0.25, though the combination of CT and Bed model provides larger (worse) brier score at year 1 to year 4 than other three models. In addition, all considered models provide good performance on the training data (c-index >70; IBS values <0.25). Among all these models, the CT only model has the worst performance with the smallest c-index and the largest IBS value. For the validation data, the BED only model and the composite model have better performance (c-index >70, and low IBS values). All these results are presented in **Table 4**.

**Table 4:** c-index and IBS score for both training and test set in the competing risk analysis.

	Training		Test	
	c-index	IBS	c-index	IBS
**BED**	73.2	0.133	70.8	0.129
**CT**	70.7	0.147	67.1	0.139
**CT-BED**	74.6	0.134	68	0.167
**Composite**	75.4	0.134	70.7	0.136

## Discussion

With technical advancements along with the rapid digitization of information, we are witnessing an exponential increase in the accumulation of data, such as medical imaging data and 3D dose distribution, in Radiation Oncology. Traditionally, these data were primarily utilized for human perception or visual representation. Our analytical capabilities for evaluating medical imaging, specifically CT and the 3D dose distribution matrix, were constrained to simplistic, 1-dimensional methodologies, such as dose-volume histogram (DVH). However, recent advancements in computational capacities and artificial intelligence technologies have revolutionized our approach, equipping us to transform data such as CT and dose matrix into structured, minable datasets. This enables the application of sophisticated data mining techniques to unearth complex patterns and associations that may be indicative of specific clinical outcomes. This transition not only enhances our ability to explore underlying mechanisms but also opens up new avenues for predictive analytics and decision-making based on comprehensive, multi-dimensional data analysis.

Owing to its distinct advantages in providing a comprehensive and quantitative representation of the radiographic phenotype of a 3D target volume (19–25), radiomics has become an active area of research for risk assessment and treatment response prediction in cancer management (26–30). However, despite its utility, radiomics remains constrained in its capacity for comprehensive treatment response assessment, particularly in Radiation Oncology. This limitation arises because radiomics focuses on extracting and analyzing features predominantly from the images. With the recent increase in the application of radiomics techniques in the 3D dose distribution domain, dosiomics features are being increasingly examined for their potential in predictive modeling. However, existing dosiomics studies (32, 39–43) have focused more on the features extracted directly from 3D dose distribution rather than their correlation, or interaction with the medical images. Logically, it is the interplay between the radiation and the underlying characteristics of irradiated tissue, as represented by medical images, that fundamentally determines the treatment outcome. Thus, a more holistic approach that incorporates both medical images and its interaction with the radiation dose is possibly essential for a more accurate and thorough assessment of treatment response.

Our study addresses this gap by incorporating 3D dose matrix into the analytical framework and calculating five distinct interaction matrices between CT and dose. These matrices are designed to potentially represent the dynamic interplay or correlation between CT and dose. This approach enhances the comprehensiveness of our analysis, potentially providing a deeper understanding of the interactions that may influence outcomes within our study’s scope. Specifically, the Entropy matrix elucidates local variations or complexity within data, where components exhibiting low entropy are more uniform compared to those with high entropy. The Jensen-Shannon divergence (JSD) matrix and the Wasserstein distance matrix both quantify the dissimilarity between CT and dose distributions in the local environment based on distinct mathematical perspectives with different emphases. Spearman’s rank correlation matrix assesses the monotonic relationship between CT and dose distributions in the local environment. By integrating these matrices into our feature mining process, we can potentially uncover additional hidden features that represent the voxelated interplay or correlation between images and dose, which might be pivotal in determining treatment outcomes. Our approach potentially allows for a more nuanced understanding of the factors influencing therapeutic efficacy. As shown in our results, the composite model, characterized predominantly by its top features from these interaction matrices, demonstrated superior performance compared to other models, indicating these interaction matrices are informative and predictive.

Although delta-radiomics (44–48) has also been utilized to evaluate treatment response in radiation oncology, it relies on analyzing temporal changes in imaging to predict clinical outcomes post-treatment. This retrospective and passive approach does not facilitate modifications or improvements to treatment plans, which are typically assessed using relatively simplistic, 1D metrics such as DVH prior to treatment delivery. Conversely, our approach, upon thorough validation, could offer physicians the ability to proactively select more effective treatment plans, potentially reducing treatment failures.

Our study has a few limitations. First, we have a relatively small sample size, and our conclusion necessitates cautious interpretation and requires validation through external datasets. The observed discrepancy in AUC values between the training set and the test set suggests that the sample size may be insufficient for robust model evaluation. Additionally, we did not observe the significant difference in AUC for the four models, which is also likely caused by our limited sample size since we only had 28 patients in the test set. Second, although we utilized the biological effective dose (BED) to account for the variations of biological effect due to various fractionations, it should be noted that voxels within the PTV were assigned an alpha-beta ratio of 10, while voxels outside the PTV were assigned a value of 3 in our calculation of the voxelated BED, as there is currently no widely accepted model for voxelated BED based on tissue electron density. Furthermore, we chose an empirical value of 10 for the alpha-beta ratio for the cancerous tissue, although this may not be the most appropriate for NSCLC, where higher values have been suggested (49, 50). In addition, applying a uniform alpha-beta ratio across all NSCLC subtypes may be overly simplistic, given the reported variations in cell survival curves among different lung cancer (51), which potentially overlooks the nuanced biological differences. Our current analyses did not evaluate the potential impact on the model performance from these variations. We did not address these considerations in our current study primarily because these specific values have not yet been widely validated and accepted in clinical settings. Third, we did not further investigate the uncertainty of the features caused by various tube current, voxel size resampling and kernel reconstruction as it has been demonstrated that the variations in tube current do not significantly affect radiomic features (52) and that radiomic features have shown no notable correlation with exposure (53). Also, since resampling minimizes the effect of voxel size on the radiomic features (53, 54), the image sets used in our study were resampled to 1 x 1 x 2 mm³. Additionally, all images were acquired using either the B31s or B31f convolution kernel from the single vendor (Siemens), and both of B31s and B31f kernel are considered as smooth kernels with limited expected difference (55). Fourth, in our analysis, we selected free-breathing CT arbitrarily without conducting an extensive comparison of potential results using average CT, given the fact that the superiority of free-breathing CT compared to average CT within the context of clinical treatment planning remains a subject for debate. Although additional features and outcomes could be explored further using average CT, we have proved our concept that integrating the interaction between dose and CT enhances prediction accuracy compared to using CT or dose alone.

Future investigations following our proof-of-concept study are still warranted in the following areas. First, our model needs to be validated, and possibly evolved, with external datasets. We also plan to perform independent tests using newer patients once the follow-up time meets our requirements as an alternative approach for further independent validation. Second, the impact of various alpha-beta ratios techniques on model performance, for biological effectiveness of SBRT or various histology subtypes, needs to be explored. Third, we also intend to initiate a clinical trial that utilizes our dosiomics model as a plan evaluation metric, in addition to the existing DVH-based metrics, to further validate or refine the model within a prospective setting. This approach potentially mitigates the inherent limitations of most radiomics studies, which primarily are retrospective and thus often fail to incorporate all necessary biological variables.

## Conclusion

Our proof-of-concept study demonstrated that a dosiomics model, which incorporates the interaction between CT and dose, has demonstrated effectiveness in predicting treatment failures following lung SBRT treatment. This model holds potential as a proactive tool for evaluating and selecting treatment plans, aimed at reducing future treatment failures.

## Data Availability

The original contributions presented in the study are included in the article/supplementary material. Further inquiries can be directed to the corresponding authors.
